# Enhancing Safety in Refractive Surgery: A Pilot Evaluation of In Vivo Confocal Microscopy

**DOI:** 10.3390/jcm14217714

**Published:** 2025-10-30

**Authors:** Dominika Janiszewska-Bil, Magdalena Kijonka, Joanna Kokot-Lesiuk, Victor Derhartunian, Anita Lyssek-Boroń, Dariusz Dobrowolski, Edward Wylęgała, Beniamin Oskar Grabarek, Katarzyna Krysik

**Affiliations:** 1 Department of Ophthalmology, St. Barbara Hospital, Trauma Centre, 41-200 Sosnowiec, Poland; anitaboron3@gmail.com (A.L.-B.); dardobmd@wp.pl (D.D.); kkrysik@gmail.com (K.K.); 2 Collegium Medicum, WSB University, 41-300 Dabrowa Gornicza, Poland; bgrabarek7@gmail.com; 3 Department of Ophthalmology, Faculty of Medicine, Academy of Silesia, 40-555 Katowice, Poland; 4 Chair and Ophthalmology Clinic Railway Hospital Katowice, Medical University of Silesia, 40-760 Katowice, Polandlek.joanna.kokot@gmail.com (J.K.-L.); wylegala@sum.edu.pl (E.W.); 5 EyeLaser Clinic, Opernring 1, 1010 Vienna, Austria; vd@eyelaser.at

**Keywords:** in vivo confocal microscopy, refractive surgery, corneal imaging, Thygeson’s keratitis, Cogan’s dystrophy

## Abstract

**Background:** In vivo confocal microscopy (IVCM) provides high-resolution corneal imaging that may enhance preoperative and postoperative assessment in refractive surgery. This pilot study aimed to evaluate the diagnostic utility of IVCM in identifying subclinical corneal abnormalities that could influence surgical qualification and outcomes. **Methods:** A total of 7 patients (3 males, 4 females; mean age 48.8 ± 14.5 years) undergoing qualification or follow-up for refractive surgery were prospectively examined between May 2021 and March 2025. Each participant underwent a comprehensive ophthalmic evaluation, including slit-lamp biomicroscopy, corneal topography, anterior segment optical coherence tomography (AS-OCT), and IVCM using the Heidelberg Retina Tomograph II with Rostock Cornea Module. Patients with prior ocular surgery, active infection, or systemic corneal disease were excluded. **Results:** IVCM revealed subtle epithelial, stromal, and endothelial abnormalities undetectable by conventional methods. Findings such as Thygeson’s keratitis, pre-Descemet’s dystrophy, and subclinical herpes simplex keratitis led to modifications of surgical plans or disqualification in selected cases. The technique also aided postoperative evaluation of epithelial–stromal interface disorders. **Conclusions:** IVCM proved to be a valuable adjunct in detecting subclinical corneal pathology, refining patient selection, and improving safety in refractive surgery. Larger multicenter studies are warranted to validate its clinical role and define standardized indications for preoperative screening.

## 1. Introduction

Refractive surgery has become a widely accepted method for correcting visual impairments such as myopia, hyperopia, and astigmatism [1]. Procedures such as Femtosecond Laser-Assisted In Situ Keratomileusis (Femto-LASIK) and Refractive Lenticule Extraction—Small Incision Lenticule Extraction (ReLEx SMILE) offer patients an effective alternative to spectacles and contact lenses, significantly improving their quality of life [2,3,4]. These advanced surgical techniques have revolutionized corneal refractive procedures by enhancing cutting precision, improving biomechanical predictability, accelerating visual recovery, and reducing the incidence of postoperative complications such as dry eye and ectasia [5]. However, the success and safety of these procedures largely depend on a thorough preoperative evaluation of the cornea [6,7,8].

Corneal pathologies—including scarring, dystrophies, metabolic changes, and inflammatory conditions—can adversely affect surgical outcomes and increase the risk of postoperative complications [9,10]. Identifying these abnormalities before surgery is crucial to ensuring optimal results and preventing vision-threatening sequelae [11]. Although slit-lamp biomicroscopy remains a fundamental tool for assessing corneal integrity, it often lacks the resolution required to detect subtle or early-stage abnormalities [12]. Confocal microscopy has emerged as an advanced diagnostic modality that allows high-resolution, in vivo imaging of the corneal microstructure [13,14]. This non-invasive technique provides detailed visualization of the corneal epithelium, stroma, and endothelium, enabling early detection of subclinical pathologies that could compromise surgical outcomes [15].

Confocal microscopy is particularly valuable in patients with a history of ocular trauma, long-term contact lens use, unexplained visual disturbances, or suspected corneal dystrophies [15]. By revealing microscopic abnormalities such as corneal nerve alterations, inflammatory cell infiltration, or subtle stromal irregularities, this technology refines patient selection criteria, informs surgical planning, and helps minimize the risk of complications such as post-LASIK ectasia or suboptimal refractive results [16,17,18,19].

The present study aims to evaluate the role of confocal microscopy in the preoperative and postoperative assessment of patients undergoing refractive surgery. By analyzing corneal abnormalities in a cohort of surgical candidates, we sought to identify potential risk factors that could influence surgical planning, patient selection, and postoperative outcomes. Understanding the microscopic features of corneal pathology may enhance diagnostic accuracy and refine treatment strategies, ultimately contributing to safer and more effective refractive procedures. Furthermore, integrating confocal microscopy into routine preoperative screening protocols may optimize patient outcomes, improve long-term visual stability, and advance the field of refractive surgery.

It should be noted that in vivo confocal microscopy (IVCM) is a contact-based imaging technique and, although generally safe, carries a minimal risk of corneal epithelial microerosions or, in rare cases, secondary infection if aseptic conditions are not strictly maintained. In this study, all examinations were performed in a controlled hospital environment by experienced clinicians using sterile techniques, and no adverse events were observed.

## 2. Materials and Methods

### 2.1. Study Design

This prospective observational case series included seven consecutive patients who underwent IVCM during their refractive surgery consultation or follow-up between May 2021 and March 2025. All examinations were conducted prospectively, meaning that IVCM imaging was performed at the time of each patient’s clinical visit rather than retrospectively from archived data.

The cohort comprised five preoperative candidates and two postoperative patients referred for IVCM assessment due to new or unexplained visual symptoms following previous refractive surgery or in preparation for planned enhancement procedures. This design allowed evaluation of the diagnostic value of IVCM in both preoperative screening and postoperative assessment contexts.

Each patient underwent a comprehensive ophthalmic assessment, including a detailed medical history obtained through interviews and review of clinical records. Clinical examinations were performed using slit-lamp biomicroscopy, and findings were documented photographically. Following standard evaluation, IVCM was conducted to further assess corneal microstructure.

Although the primary focus was preoperative assessment, one postoperative case (Patient 6) was included to demonstrate the utility of IVCM in detecting delayed epithelial and stromal interface complications after laser refractive surgery. Inclusion of this case highlights the broader applicability of IVCM, not only for preoperative screening but also for diagnosing postoperative structural abnormalities that may affect visual stability and patient satisfaction.

All participants provided written informed consent prior to enrollment after receiving a detailed explanation of the study’s objectives, procedures, and potential risks.

During the recruitment period (May 2021–March 2025), 28 consecutive patients were offered IVCM as part of their refractive surgery qualification or postoperative evaluation. Of these, seven patients (25%) provided written informed consent and completed the examination. The remaining 21 declined participation, citing the contact-based nature of the procedure, time constraints, or anticipated discomfort. This acceptance rate reflects both the limited availability of IVCM and the selectivity of patients willing to undergo detailed microstructural assessment, introducing a potential selection bias acknowledged in the interpretation of results.

Given the specialized nature of this study, the sample size was intentionally limited. In clinical practice, patients with significant corneal abnormalities are usually excluded from refractive surgery at earlier qualification stages. This study therefore focused on a subgroup of motivated patients who remained eligible for refractive procedures despite subtle or borderline findings. Moreover, as IVCM is not routinely performed during standard preoperative evaluations, its use was limited to specialized hospital-based settings requiring patient cooperation for a time-consuming, mildly invasive examination. These factors collectively contributed to the small study population, which nonetheless yielded valuable clinical insights.

### 2.2. Patient Data

This prospective study included seven patients evaluated for refractive surgery at our ophthalmology departments. All participants provided written informed consent before enrollment. Inclusion criteria encompassed individuals with myopia, hyperopia, or astigmatism who were candidates for laser vision correction procedures, including Femto-LASIK, ReLEx SMILE, or phakic intraocular lens (ICL) implantation.

Patients with a history of ocular surgery, active ocular infection, or systemic conditions affecting the cornea were excluded. Each participant underwent a comprehensive ophthalmic evaluation including best-corrected visual acuity (BCVA), manifest and cycloplegic refraction, slit-lamp biomicroscopy, corneal topography (Pentacam, Oculus Optikgeräte GmbH, Wetzlar, Germany), and pachymetry. Additional diagnostic tests included anterior segment optical coherence tomography (AS-OCT, Triton, Topcon, Warsaw, Poland) to assess corneal morphology, epithelial thickness mapping, and endothelial cell density measurement using specular microscopy.

### 2.3. IVCM (HRT2-RCM)

IVCM was performed using the Heidelberg Retina Tomograph II (HRT II) equipped with the Rostock Cornea Module (Heidelberg Engineering GmbH, Heidelberg, Germany). To ensure optimal image quality and prevent air bubble formation, a generous amount of Comfort Gel ophthalmic ointment (Bausch & Lomb GmbH, Berlin, Germany) was applied to the front surface of the microscope lens. A Tomo-Cap (Heidelberg Engineering GmbH) was securely mounted on the lens holder to maintain stable and uniform contact with the corneal surface.

Both the central and peripheral regions of the cornea were systematically scanned layer by layer. The HRT2-RCM utilizes a 360° water-immersion objective lens (Olympus Europa GmbH, Hamburg, Germany) and a 670 nm diode laser light source. The system captures 384 × 384-pixel images over a 400 × 400 µm field of view. Each eye was examined twice during the patient’s initial visit to ensure the consistency and accuracy of imaging results.

In addition to qualitative evaluation, a semi-quantitative assessment was performed using established parameters described in the literature. Corneal nerve fiber density (CNFD) and corneal nerve fiber length (CNFL) were estimated based on standardized confocal microscopy reference images, while keratocyte reflectivity was graded on a 0–3 scale (0 = absent, 1 = mild, 2 = moderate, 3 = marked) according to Hovakimyan et al. [20]. Hyper-reflective deposits, interface disruptions, and nerve irregularities were analyzed in at least five representative frames per corneal layer. Although the small sample size precluded statistical testing, these standardized grading approaches facilitated intra-case comparisons and ensured reproducibility of descriptive findings.

## 3. Results

This study included seven patients (four females, three males) who underwent comprehensive ophthalmic evaluation and in vivo confocal microscopy (IVCM) either prior to or following refractive surgery qualification. Confocal microscopy significantly influenced clinical decision-making by revealing subclinical abnormalities or confirming diagnoses that affected surgical eligibility or the choice of procedure.

**Figure 1:**
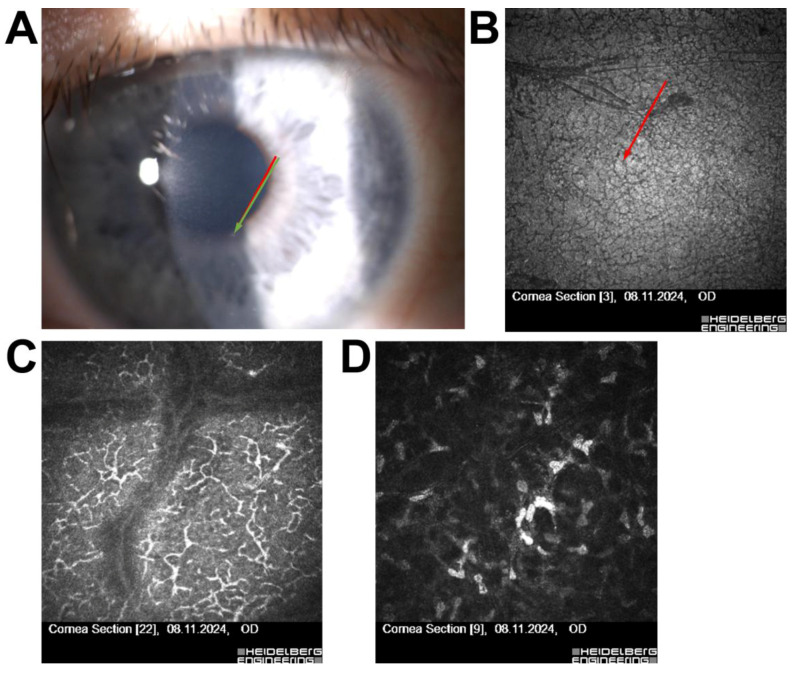
(**A**) Slit-lamp image of the right eye (OD) showing a bright subepithelial lesion without anterior chamber reaction. The red arrow indicates the central subepithelial punctate opacity, while the green arrow highlights an additional peripheral punctate epithelial lesion. (**B**) IVCM image of the Bowman’s layer and superficial epithelium (red arrow: focal epithelial hyper-reflectivity). (**C**) IVCM image at the level of the sub-basal nerve plexus showing irregular, fragmented nerve fibers. (**D**) IVCM image of the anterior stroma showing preserved stromal architecture. All IVCM images were acquired using the Heidelberg Retina Tomograph II–Rostock Cornea Module (field of view: 400 × 400 µm; optical magnification ×800; scale bar = 50 µm).

Patient 1—A 52-year-old female with a history of long-term contact lens use presented with punctate epithelial opacities. IVCM revealed subepithelial hyper-reflective deposits and irregularities in the subnasal nerve plexus, findings consistent with Thygeson’s superficial punctate keratitis (TSPK). These abnormalities, together with her clinical symptoms, led to disqualification from refractive surgery due to the increased risk of postoperative complications and recurrence of epithelial lesions (Figure 1).

**Figure 2:**
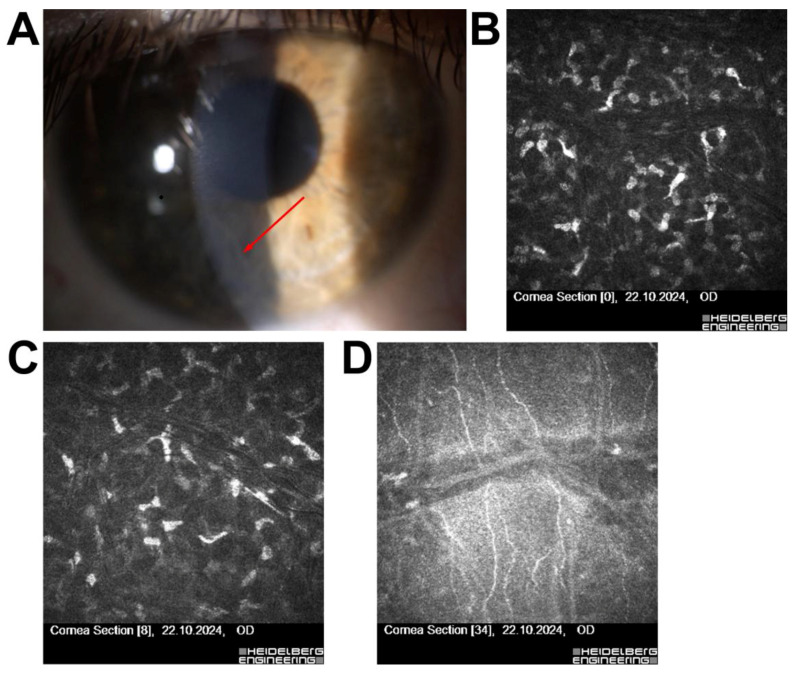
Multimodal corneal imaging in a patient with suspected corneal pathology. (**A**) Slit-lamp photograph of the left eye (OS) showing linear pigment deposition (red arrow). (**B**) IVCM of the superficial epithelium, (**C**) sub-basal nerve plexus, and (**D**) endothelium, all showing normal morphology and intact cellular patterns. All IVCM images were acquired using the Heidelberg Retina Tomograph II–Rostock Cornea Module (field of view: 400 × 400 µm; optical magnification ×800; scale bar = 50 µm).

Patient 2—A 30-year-old female with high myopia and astigmatism exhibited a Hudson–Stähli line during slit-lamp examination—an iron deposition line typically regarded as benign. Although IVCM did not reveal any stromal or endothelial abnormalities, the patient was disqualified from surgery due to suspected corneal biomechanical instability, which posed an elevated risk of postoperative ectasia (Figure 2).

**Figure 3:**
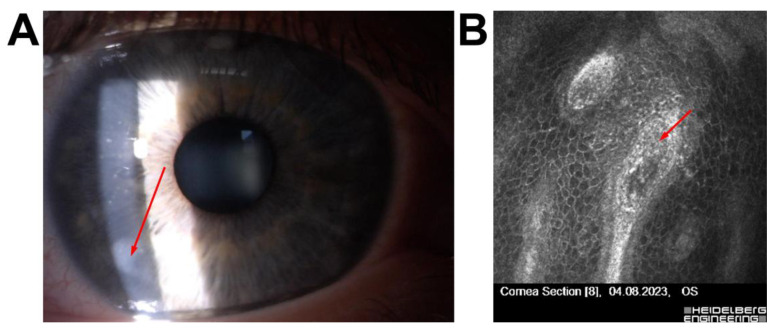
IVCM image of the cornea in a patient with a history of corneal foreign body. (**A**) IVCM image of the anterior stroma showing a sharply demarcated corneal scar after a foreign body (red arrow); (**B**) mid-stromal layer demonstrating absence of active inflammation (red arrow). All IVCM images were acquired using the Heidelberg Retina Tomograph II–Rostock Cornea Module (field of view: 400 × 400 µm; optical magnification ×800; scale bar = 50 µm).

Patient 3—A 49-year-old female with a remote history of corneal foreign body removal underwent IVCM, which confirmed a sharply demarcated stromal scar without evidence of active inflammation or keratocyte activation. The absence of subclinical pathology indicated a low surgical risk, and the patient was qualified for laser vision correction (Figure 3).

**Figure 4:**
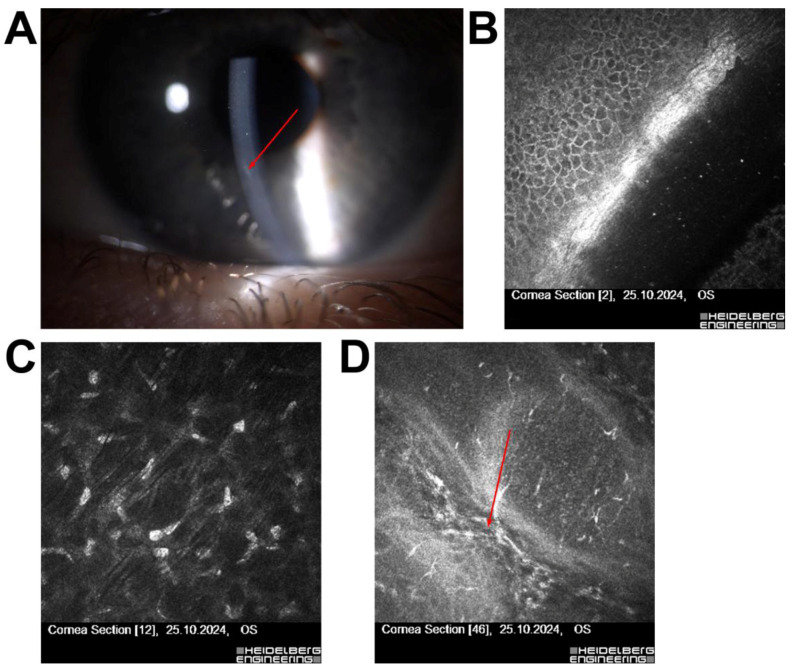
Corneal imaging in a patient with a superficial corneal scar qualified for ReLEx SMILE (**A**) Slit-lamp photograph showing a linear corneal scar visible in biomicroscope (red arrow); (**B**) IVCM of the epithelium and Bowman’s layer confirming absence of deeper stromal involvement. (**C**) IVCM at the level of the anterior stroma showing normal stromal keratocyte morphology without fibrosis. (**D**) IVCM of the mid-stroma revealing well-organized stromal collagen fibers with no evidence of scarring deeper in the cornea (red arrow). All IVCM images were acquired using the Heidelberg Retina Tomograph II–Rostock Cornea Module (field of view: 400 × 400 µm; optical magnification ×800; scale bar = 50 µm).

Patient 4—A 29-year-old male scheduled for ReLEx SMILE had a faint superficial corneal scar identified during slit-lamp examination. IVCM confirmed that the lesion was confined to the epithelial layer, with no involvement of the stroma or signs of inflammation. The surgery was successfully performed without complications (Figure 4).

**Figure 5:**
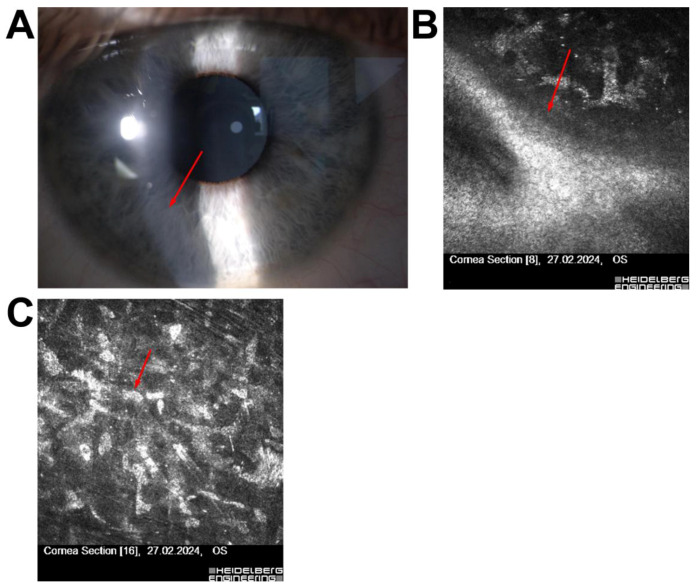
IVCM findings in a patient with suspected pre-Descemet’s dystrophy and low corneal thickness. (**A**) Slit-lamp photograph showing punctate endothelial changes (red arrow). (**B**,**C**) IVCM images of the posterior stroma and Descemet’s membrane, showing thickened pre-Descemet’s region characteristic of pre-Descemet’s dystrophy (red arrow). All IVCM images were acquired using the Heidelberg Retina Tomograph II–Rostock Cornea Module (field of view: 400 × 400 µm; optical magnification ×800; scale bar = 50 µm).

Patient 5—A 54-year-old female with high myopia, reduced corneal thickness, and a large pupil was diagnosed with pre-Descemet’s corneal dystrophy based on IVCM findings. Hyper-reflective stromal deposits were observed in the absence of endotheliitis. Due to insufficient stromal thickness for laser ablation, phakic intraocular lens (ICL) implantation was proposed. The patient was counseled regarding possible night vision disturbances associated with her large scotopic pupil (Figure 5).

**Figure 6:**
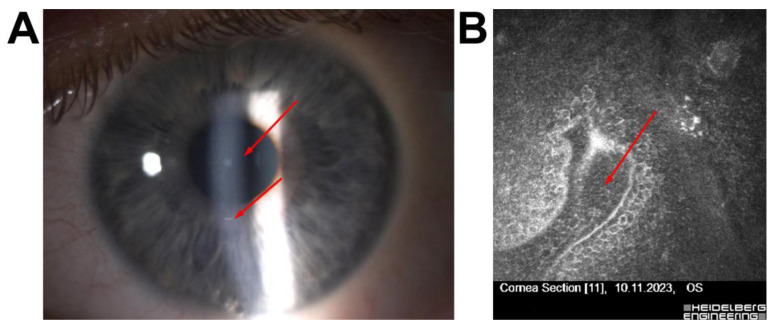
Corneal imaging in a postoperative patient with secondary Cogan’s dystrophy following Femto-LASIK (**A**) Slit-lamp photograph showing spot thickening of the epithelium visible in biomicroscope (red arrow); (**B**) IVCM image of the epithelial–stromal interface demonstrating epithelial folds and basement membrane irregularities typical of secondary Cogan’s dystrophy; the red arrow indicates a focal area of basement membrane disruption with hyper-reflective epithelial changes. All IVCM images were acquired using the Heidelberg Retina Tomograph II–Rostock Cornea Module (field of view: 400 × 400 µm; optical magnification ×800; scale bar = 50 µm).

Patient 6—A 56-year-old male with a history of Femto-LASIK three years earlier presented with fluctuating visual acuity. IVCM revealed epithelial–stromal interface irregularities, including basement membrane disruption and epithelial cell disarray, indicative of secondary Cogan’s dystrophy. Based on these findings, phototherapeutic keratectomy (PTK) was performed instead of flap-lift enhancement, resulting in improved uncorrected visual acuity (Figure 6).

**Figure 7:**
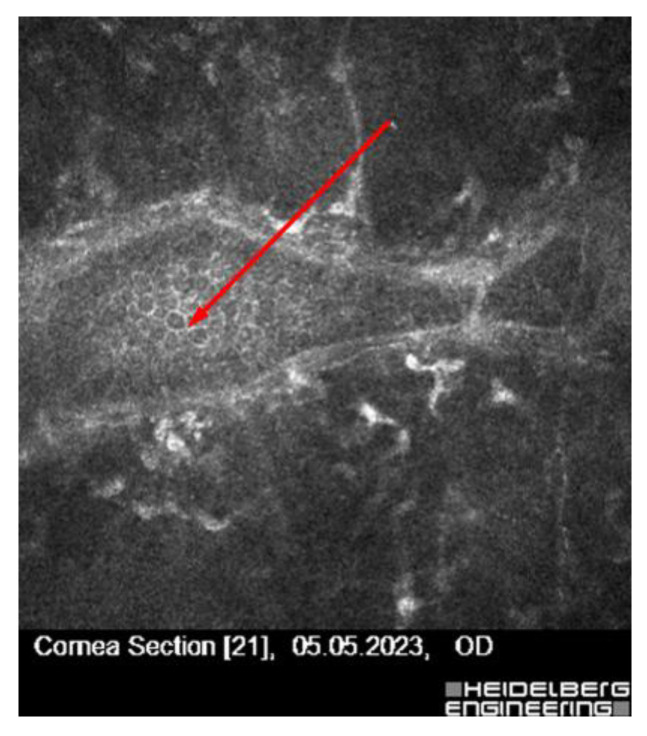
IVCM finding in an asymptomatic patient with subclinical HSK. The image shows hyperreflective activated keratocytes and alterations in the sub-basal nerve plexus consistent with subclinical HSK (red arrow). The IVCM image was acquired using the Heidelberg Retina Tomograph II–Rostock Cornea Module (field of view: 400 × 400 µm; optical magnification ×800; scale bar = 50 µm).

Patient 7—A 58-year-old asymptomatic male presented with a subtle subepithelial lesion. IVCM demonstrated early features of subclinical herpes simplex keratitis (HSK), including activated keratocytes and increased stromal reflectivity. Although asymptomatic, the patient received prophylactic oral acyclovir before surgery to reduce the risk of postoperative viral reactivation (Figure 7).

A summary of patient demographics, clinical histories, IVCM findings, and surgical outcomes is presented in Table 1.

For clarity, hyper-reflective deposits were defined as discrete, bright stromal or subepithelial foci exhibiting > 30% higher reflectivity than the surrounding keratocytes within the same frame. Nerve irregularities referred to disorganization or fragmentation of the sub-basal nerve plexus, while interface disruptions denoted discontinuities or folds at the epithelial–stromal boundary.

The mean corneal nerve fiber length (CNFL) across the analyzed eyes was 16.2 ± 3.1 mm/mm^2^, and the average keratocyte density in unaffected stromal regions was 978 ± 110 cells/mm^2^, consistent with published normative values. These semi-quantitative findings corroborate the qualitative interpretation of microstructural alterations detected by IVCM.

## 4. Discussion

IVCM has proven to be a valuable tool for the detailed assessment of corneal microstructure, providing high-resolution imaging of the epithelial, stromal, and endothelial layers [15]. This study demonstrated its clinical utility in both pre- and postoperative evaluations of refractive surgery candidates by identifying subtle corneal abnormalities undetectable through conventional examination methods. Our findings align with previous research, confirming that IVCM can reveal subclinical pathological changes—such as inflammatory infiltrates, nerve plexus alterations, and metabolic deposits—thereby improving surgical planning and risk assessment. Although this was a prospective study, the inclusion of postoperative and subclinical cases reflects real-world clinical practice, in which IVCM was prospectively applied to all referred patients, regardless of whether they were first-time surgical candidates or had undergone prior refractive procedures. This approach highlights the broader diagnostic relevance of IVCM beyond a purely preoperative context.

While this pilot study did not include a direct quantitative comparison between slit-lamp examination alone and slit-lamp assessment combined with IVCM, our observations illustrate how IVCM provided additional diagnostic insights that refined clinical judgment in several cases. The technique revealed microstructural alterations invisible under slit-lamp biomicroscopy, allowing for more confident qualification or disqualification of candidates. Future prospective studies with controlled, comparative designs are warranted to objectively quantify the diagnostic yield and clinical impact of IVCM relative to standard preoperative modalities [17,19].

A representative case is Patient 1, a 52-year-old female with long-term contact lens use, initially suspected of having adenoviral keratitis. IVCM confirmed the diagnosis of Thygeson’s superficial punctate keratitis (TSPK), a rare, noninfectious condition characterized by bilateral, recurrent punctate epithelial opacities without stromal involvement [21,22,23,24]. Prior studies, including those by Kobayashi et al., have described reflective epithelial deposits and subepithelial haze as characteristic IVCM findings in TSPK [17]. These characteristic IVCM features were also observed in our case, confirming the diagnosis and justifying disqualification from refractive surgery. This finding highlights the pivotal role of IVCM in resolving ambiguous or overlapping clinical presentations, particularly when slit-lamp or topographic findings are inconclusive [25].

In Patient 2, a 30-year-old female with high myopia and astigmatism, slit-lamp biomicroscopy revealed a Hudson–Stähli line—an iron deposition within the corneal epithelium typically considered benign [26,27]. IVCM confirmed a normal corneal microstructure, indicating no stromal or endothelial pathology. The patient was disqualified from surgery not due to IVCM findings, which were unremarkable, but because of corneal biomechanical risk factors detected during topography and pachymetry. Thus, IVCM played a confirmatory role, ruling out microstructural pathology rather than directly determining surgical eligibility [28,29].

Patient 3, with a history of prior corneal foreign body removal, demonstrated a well-demarcated stromal scar without signs of active inflammation on IVCM. This finding supported surgical qualification and reinforced earlier evidence that confocal microscopy can distinguish quiescent stromal scarring from subclinical inflammation or keratocyte activation [20].

In Patient 4, a 29-year-old female scheduled for ReLEx SMILE, slit-lamp examination raised concern due to a superficial corneal scar. IVCM confirmed that the lesion was confined to the epithelial layer, with no stromal involvement or inflammatory changes. The surgery was subsequently performed without complications, demonstrating how IVCM can clarify the clinical relevance of corneal scars by differentiating benign surface irregularities from deeper lesions that may compromise refractive outcomes [28,30].

Patient 5, a 54-year-old female with high myopia and reduced corneal thickness, was diagnosed with pre-Descemet’s corneal dystrophy based on IVCM findings, which showed hyper-reflective stromal deposits in the absence of endotheliitis. This confirmation supported the choice of phakic intraocular lens (ICL) implantation, as corneal thickness was insufficient for laser ablation [31]. The patient’s large scotopic pupil required preoperative counseling regarding potential night vision disturbances, although it did not alter the procedural plan [32].

A postoperative case, Patient 6, involved a 56-year-old male who had undergone Femto-LASIK three years earlier and presented with fluctuating visual acuity. IVCM revealed epithelial–stromal interface irregularities, including basement membrane disruption and epithelial disarray, consistent with secondary Cogan’s dystrophy [33]. Based on these findings, PTK was performed instead of flap-lift enhancement, resulting in improved uncorrected visual acuity [34,35].

This case underscores the utility of IVCM in postoperative settings, where it aids in differentiating dystrophic or structural interface abnormalities from inflammatory or infectious causes of visual fluctuation [28].

Finally, Patient 7, an asymptomatic 58-year-old male with a subtle subepithelial lesion, exhibited IVCM findings consistent with subclinical herpes simplex keratitis (HSK), including activated keratocytes and increased stromal reflectivity. Although clinically silent, the patient received prophylactic oral acyclovir prior to surgery to mitigate the risk of postoperative viral reactivation [36].

Because the risk of postoperative corneal ectasia is primarily governed by corneal biomechanical stability, integrating IVCM with quantitative biomechanical assessments—such as Corvis ST dynamic Scheimpflug imaging or the Ocular Response Analyzer—could yield a more comprehensive preoperative risk profile. These instruments were unavailable during the present study, representing a methodological limitation. Future research protocols should combine IVCM-derived microstructural parameters with biomechanical indices such as deformation amplitude, corneal hysteresis, or stiffness parameter at first applanation to enhance predictive modeling and individualized risk stratification for refractive surgery candidates [37,38].

Based on our observations and prior literature, we propose that IVCM should be selectively employed in patients with: (1) a history of long-term contact lens wear or recurrent keratitis; (2) borderline corneal thickness or irregular topography; (3) unexplained visual symptoms incongruent with slit-lamp or topographic findings; or (4) suspected early dystrophic, inflammatory, or viral changes. Incorporating IVCM at these critical decision points may help identify subclinical contraindications and enhance surgical safety. Although IVCM systems require substantial investment and operator expertise, their implementation may be justified in tertiary or referral centers where refractive surgery volume and diagnostic precision directly affect patient safety. In non-tertiary settings, regional collaboration or referral for IVCM imaging could optimize resource utilization while maintaining safety standards.

This study has several limitations. First, the small and heterogeneous cohort (n = 7) limits statistical power and generalizability. Second, although all patients were examined prospectively, inclusion of both preoperative and postoperative cases introduces clinical heterogeneity. Third, no longitudinal follow-up data on postoperative outcomes were collected. Fourth, the study was conducted at a single tertiary center without a control group, and corneal biomechanical testing (Corvis ST/ORA) was unavailable. Finally, the limited acceptance rate for IVCM introduces potential selection bias. These factors collectively constrain the strength of the conclusions and should be addressed in future research.

To validate these preliminary findings, larger multicenter studies with standardized protocols are needed. Future investigations should employ harmonized confocal scoring systems, integrate biomechanical and topographic indices, and include longitudinal follow-up of visual and refractive outcomes. Such studies will help determine the predictive value of IVCM for surgical safety and its potential incorporation into evidence-based preoperative screening algorithms.

## 5. Conclusions

The findings of this study underscore the diagnostic value of IVCM in both the preoperative and postoperative evaluation of refractive surgery candidates. By enabling detailed visualization of microscopic structural alterations—such as subclinical inflammation, epithelial–stromal interface irregularities, and metabolic or dystrophic changes—IVCM provides critical information often undetectable through standard examination techniques. This enhanced diagnostic precision supports more accurate patient selection, facilitates individualized surgical planning, reduces postoperative risk, and ultimately contributes to improved visual outcomes. Although not intended for routine use in all candidates, IVCM serves as an important adjunct in cases with ambiguous or borderline findings.

This pilot study was limited by its small sample size and lack of quantitative comparison. Future studies involving larger, standardized cohorts are warranted to assess its cost-effectiveness, clinical impact, and potential integration into comprehensive preoperative screening algorithms for refractive surgery.

## Figures and Tables

**Table 1:** Summary of Patient Characteristics, Confocal Microscopy Findings, and Surgical Outcomes.

Patient	Age/Sex	Clinical History	Confocal Microscopy Findings	Diagnosis	Clinical Decision/Outcome
1	52/F	Long-term contact lens use	Subepithelial opacities	Thygeson’s superficial punctate keratitis	Disqualified from surgery
2	30/F	High myopia, astigmatism	Hudson–Stähli line (iron deposition)	Benign corneal iron line	Disqualified due to ectasia risk
3	49/F	Corneal foreign body removal (remote)	No active inflammation	Low ectasia risk	Qualified for surgery
4	29/M	Scheduled for ReLEx SMILE	Superficial corneal scar, no stromal/inflammatory changes	Scar without contraindications	Surgery completed without complications
5	54/F	High myopia, low corneal thickness, large pupil	Pre-Descemet’s corneal dystrophy, no endotheliitis	Dystrophy	Qualified for phakic ICL implantation
6	56/M	Post-Femto-LASIK (3 years), fluctuating vision	Features of Cogan’s dystrophy	Secondary Cogan’s dystrophy	Treated with PTK (20 µm), UCVA improved
7	58/M	Asymptomatic, subepithelial lesion	Subclinical herpes simplex keratitis	Herpes simplex (subclinical)	Antiviral prophylaxis (acyclovir) before planned surgery

F, female; M, male.

## Data Availability

All data generated or analyzed during this study are included in this published article.

## Abbreviations

The following abbreviations are used in this manuscript:

BCVA	Best-Corrected Visual Acuity
Femto-LASIK	Femtosecond Laser-Assisted In Situ Keratomileusis
HSK	Herpes Simplex Keratitis
ICL	Implantable Collamer Lens (Phakic Intraocular Lens)
IVCM	In Vivo Confocal Microscopy
OCT	Optical Coherence Tomography
OD	Oculus Dexter (Right Eye)
OS	Oculus Sinister (Left Eye)
PTK	Phototherapeutic Keratectomy
SMILE	Small Incision Lenticule Extraction
TSPK	Thygeson’s Superficial Punctate Keratitis
